# The OSR9 Regimen: A New Augmentation Strategy for Osteosarcoma Treatment Using Nine Older Drugs from General Medicine to Inhibit Growth Drive

**DOI:** 10.3390/ijms242015474

**Published:** 2023-10-23

**Authors:** Richard E. Kast

**Affiliations:** IIAIGC Study Center, Burlington, VT 05408, USA; richarderickast@gmail.com

**Keywords:** chemotherapy, osteosarcoma, repurposing

## Abstract

As things stand in 2023, metastatic osteosarcoma commonly results in death. There has been little treatment progress in recent decades. To redress the poor prognosis of metastatic osteosarcoma, the present regimen, OSR9, uses nine already marketed drugs as adjuncts to current treatments. The nine drugs in OSR9 are: (1) the antinausea drug aprepitant, (2) the analgesic drug celecoxib, (3) the anti-malaria drug chloroquine, (4) the antibiotic dapsone, (5) the alcoholism treatment drug disulfiram, (6) the antifungal drug itraconazole, (7) the diabetes treatment drug linagliptin, (8) the hypertension drug propranolol, and (9) the psychiatric drug quetiapine. Although none are traditionally used to treat cancer, all nine have attributes that have been shown to inhibit growth-promoting physiological systems active in osteosarcoma. In their general medicinal uses, all nine drugs in OSR9 have low side-effect risks. The current paper reviews the collected data supporting the role of OSR9.

## 1. Introduction

**Preface:** *In trying to hold onto everything, one holds onto nothing.*

King Fredrick the Great, 1712–1786

As things stand in 2023, metastatic osteosarcoma (OS) is often fatal within a few years of diagnosis. Extensive local bone destruction, aggressive surrounding soft tissue infiltration, osteoid deposition, and early metastases to the lungs are characteristic features of OS.

Predisposing factors are preexisting Paget’s disease or a prior cancer. The OS primary tumor is highly infiltrative. Pulmonary metastases are frequent and worsen prognosis. Although still not a common tumor, its incidence has been steadily increasing over recent decades. There has been little progress in improving survival in metastatic OS in the last 40 years, and five-year survival remains at ~30% [1]. OSR9 is a new approach to this problem. This paper presents the data outlining the rationale behind OSR9 treatment and for using a repurposed multidrug adjunctive regimen generally by adding it to current treatment options in metastatic OS.

The OSR9 regimen uses nine non-oncology drugs that have been repurposed as adjuncts to standard current OS treatments: (1) the anti-nausea drug aprepitant, (2) the analgesic drug celecoxib, (3) the anti-malaria drug chloroquine, (4) the anti-malaria drug dapsone, (5) the alcoholism treatment drug disulfiram, (6) the antifungal drug itraconazole, (7) the anti-diabetes drug linagliptin, (8) the antihypertension drug propranolol, and (9) the psychiatric drug quetiapine. These drugs and the data showing evidence of their ability to interfere with OS growth are discussed in detail below. See Table 1 for some of the core features of these OSR9 drugs’ attributes that are relevant in treating OS.

None of the OSR9 drugs have been proven to be of benefit during treatment for OS or any other cancer. However, since most physicians, regardless of specialty, are familiar with their use in their approved, non-oncology indications, all these OSR9 drugs are generally well-tolerated, and all nine drugs have preclinical evidence that they might retard OS growth, and so the risk/benefit ratio would favor a small study on this OSR9 regimen in metastatic OS patients for whom no other treatment options exist.

On X-ray plain films, OS displays the following features:

- Patchy cortical bone destruction
- Areas of sclerotic bone
- Wide and gradual transitions from normal-appearing trabecular bone to clearly abnormal, moth eaten, and irregularly mineralized bone
- Peritumoral soft tissue edema
- Codman’s triangle (irregular periosteum that has lifted off where advancing tumor margins destroy periosteal new bone formation faster than it can ossify)

OS is a tumor composed of mesenchymal cells held at an immature osteoblast differentiation stage. Local regions of genome hypermutations (kataegis) are present [2,3,4]. These regions of grossly disturbed genome show ploidy increases and decreases and chromosomal amplifications, losses, gains, and mutations. These genomic abnormalities are not stable either; they change rapidly. *RUNX2*, essential for the normal differentiation of bone and mammary tissues, have elevated copy numbers, RNA, and protein levels in OS tumors, but these are ineffective in driving differentiation as RUNX-2 would normally do in normal bone [2,3,4,5].

## 2. The Need for Both Repurposed and Multidrug Regimens in OS

There has been little progress in treating OS over the last 30+ years despite dozens of clinical trials of combinations of new and older cytotoxic drugs, tyrosine kinase receptor inhibitors, and monoclonal antibodies directed at putative growth factors [3,6,7]. A large peak OS incidence occurs between 5 and 25 years of age and a lesser peak incidence occurs between 75 and 85, with significantly poorer survival in the older group [6,7]. Overall, the five-year survival for OS in 0 to 25 year-olds was 36%; for 25 to 59 year-olds, it was 15%; and for >60 year-olds, it was 6% [6].

Twelve recent papers have presented the thinking about a new school of cancer treatment that espouses adding multiple repurposed non-oncology drugs to standard current cytotoxic chemotherapies. A series of recent papers from the IIAIGC Study Center [8,9,10,11] and from other centers [12,13,14,15,16,17,18,19] have outlined this reasoning for why such a multidrug regimen would be needed to treat common deadly metastatic cancers. Briefly summarized, the 12 papers outline these reasons as follows:

### 2.1. Why Many Drugs Are Needed

1. Absent a “silver bullet”, many drugs may be needed to address tumors’ readily evolving and multiple resistance pathways and their ability to shift reliance to alternate growth drives when the one in use becomes blocked.
2. The common metastatic deadly cancers have metabolic flexibility, shifting between aerobic oxphos, aerobic glycolysis, anaerobic glycolysis, beta-oxidation, autophagy, glutamine, and other energy sources as circumstances dictate.
3. The common metastatic deadly cancers are hardy tissues with growth drive flexibility. The many alternate growth driving pathways can cross-cover for any one or two that become blocked.
4. Inherent multiple subpopulations exist in these cancers, even before any treatment. Each subpopulation has its own particular set of growth drives and inhibition susceptibilities and resistances. Treatment-induced selection pressure favors the development of resistant clones.
5. Ionizing irradiation and many common cytotoxic chemotherapies that are currently used to treat cancer are mutagens that hasten responses to selection pressure.

### 2.2. Why Repurposed Non-Oncology Drugs Are Needed

1. We are constrained in treating today’s illnesses with today’s tools.
2. Directly cytotoxic and genotoxic drugs have limits on how many, how much, and how often they can be used without destroying bone marrow or other essential body systems.
3. Cancers, generally, and OS, specifically, use normal physiological growth drive pathways, but they pathologically and exaggeratedly engage them. We have many drugs in general medical practice with established safety for influencing those core mammalian systems.
4. Repurposed drugs from general medical practice are cheap, generic, readily available, and well-tolerated, and GPs worldwide are already familiar with their use.

## 3. The OSR9 Drugs

The drugs in the OSR9 regimen are reviewed below. It might be thought of as curious that two of these repurposed drugs—chloroquine and dapsone—have decades of use in treating malaria and a third drug, itraconazole, is cytotoxic to *Plasmodium falciparum* in vitro at 1 microg/mL [20].

That three of the nine OSR9 drugs are cytotoxic to a protozoan becomes less strange in light of 2.2 number 3 above. Cancers, generally, and OS, specifically, use the same physiological systems that non-malignant tissues use in their day-to-day functioning. There is great commonality in physiological systems among Eukaryotes. They generally use the same or similar physiological systems.

Also not strange is the fact that the individual OSR9 drugs each have several seemingly unrelated effects on different cell physiological systems. Such many-target attributes of common drugs are common.

### 3.1. The Anti-Nausea Drug Aprepitant

Aprepitant is a generic, anti-nausea drug approved for use in treating cancer chemotherapy-related nausea and vomiting. It inhibits the NK-1 receptor for substance P (also called NK-1), an 11 amino-acid peptide [21,22]. Hundreds of clinical studies have attested to the safety and effectiveness of aprepitant in treating chemotherapy-related nausea and vomiting. Aprepitant is experiencing a resurgence of interest as an adjunct to retard growth across a variety of cancers [23,24,25,26,27]. Aprepitant has an eminently benign side effect profile.

In the past decade, dozens of papers have attested to the expression of, and growth-metastasis drive contributions from, the NK-1 receptor across a variety of the common cancers [28,29,30,31,32,33,34,35,36,37,38,39]. In the past decade, dozens of papers have shown that aprepitant mediates the in vitro arrest in growth and migration across the common human cancers [23,25,40,41,42,43,44,45,46,47,48,49,50,51,52].

Specifically in OS, recent reviews have presented the rationale for using aprepitant to retard growth [53,54,55]. OS cells express NK-1 mRNA and the NK-1 receptor, suggesting an autocrine growth drive [53,54,55,56]. Aprepitant inhibited the growth of OS in a murine xenotransplant model [53].

Despite this wealth of evidence that NK-1/substance P signaling forms a growth-driving element across a wide variety of cancers, aprepitant’s only clinical use so far has been its use in treating glioblastoma where preliminary evidence indicates a survival advantage from its use in the ten-drug CUSP9v3 protocol for treating glioblastoma [9].

### 3.2. The Analgesic Celecoxib

Celecoxib is a cyclooxygenase (COX) inhibitor selective for the COX-2 isoform. Celecoxib is commonly used to treat pain of diverse origins [57,58]. It lacks the platelet aggregation inhibitory activity seen with some other COX inhibitors [59], and it does not have the potential to create the gastric ulcerations that are associated with other COX inhibitors [60,61].

Shortly after introduction to clinical practice in the symptomatic treatment of pain, anti-cancer effects were noted both empirically and by theoretical reasoning [62,63]. Celecoxib is now being widely studied and even used on and off cancer treatment protocols in a variety of different cancers [64,65,66,67,68,69,70,71]. Celecoxib has demonstrated preclinical synergy with a variety of other traditionally used cytotoxic drugs in suppressing OS growth [72,73,74,75].

COX is a sine qua non enzyme in the synthesis of prostaglandin E. The COX-2 isoform is widely recognized as being upregulated, forming one of the many upregulated and pathophysiological-driving elements across the common cancers [68,76,77,78,79,80]. Specifically in OS, COX-2 overactivity has been repeatedly demonstrated in both pediatric and adult cases [81,82,83,84,85,86,87]. The degree of COX-2 immunohistochemical expression is inversely associated with OS patient survival duration, implying a growth-promoting role for COX-2 [86].

Human OS tissues heavily immunostain positive for COX-2 [88]. COX-2 has been demonstrated to be an in vitro driver of OS proliferation, migration, and cell survival [89]. Of note, celecoxib must be dosed at a minimum of 600 mg p.o. bid for the adequate inhibition of COX-2 when treating any cancer [90]. Lower doses are effective when treating pain.

Carbonic anhydrase (CA) catalyzes the reaction as follows:

CO_2_ + H_2_O **→** H^+^ and bicarbonate.

CA has 12 isoforms, some are soluble in cytoplasm (CAII), while some are transmembrane (CAIX and CAXII. The net effect is the further acidification of the extracellular milieu. OS, similar to other cancers, grows with a disorganized architecture, abnormally functioning, malformed vasculature, and a stroma that impedes diffusion. CO_2_ (pKa 6.35) has less of an acidifying effect than lactic acid d (pKa 3.86) does. CO_2_ freely diffuses across outer cell membranes, bicarbonate and protons do not.

**Figure 1:**
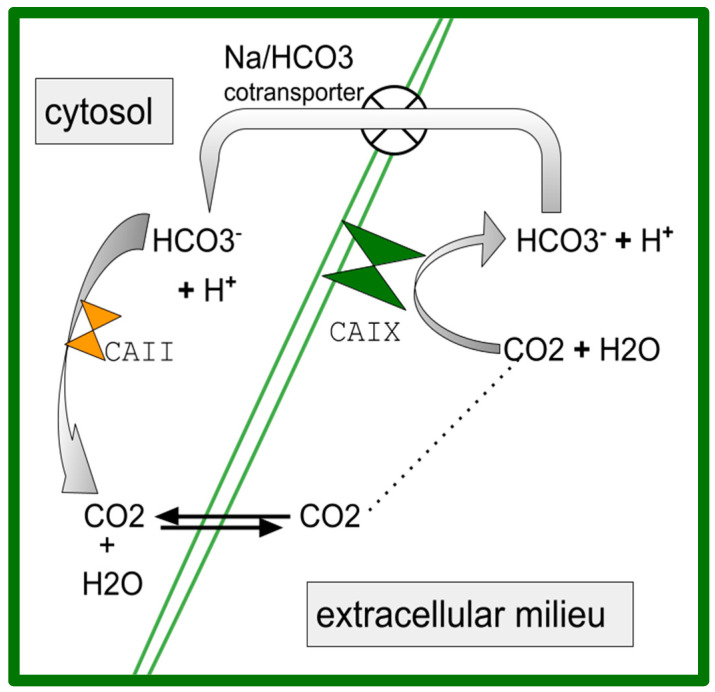
A simplified schematic of the role of carbonic anhydrase (CA) in acidifying tumor extracellular space and maintaining a relatively basic cytosol [107]. CAIX is outer membrane bound acting in the extracellular space while CAII is cytosolic. Both are inhibited by celecoxib (references in Section 3.2).

Celecoxib also inhibits CA [91,92,93]. There is a particularly important role for dual COX-2/CA inhibitors in the treatment of cancers generally [94,95]. CAIX participates in acidification of the extracellular tumor milieu, with maintenance of an alkaline intracellular milieu with an enhanced growth vigor consequent to this proton efflux across a wide variety of common human cancers [96,97,98,99,100]. Also in OS, CA-mediated cellular proton efflux is an important homeostasis-maintaining accommodation to the malignancy-characteristic aerobic glycolysis that is present in OS [101,102,103,104,105,106]. Celecoxib has the potential to undermine this malignancy-related homeostatic pathway. Figure 1 shows the basic pathway of how CAII and CAIX mediate proton export and the maintenance of relatively alkaline cytosol [107].

Celecoxib performs the nanoM inhibition of CAIX [103]. CAIX participates in creating the characteristic OS migration, survival, and acidification of the extracellular milieu [108,109,110,111]. Higher CAIX expression is associated with shorter OS survival [112], which we also see in pancreatic ductal adenocarcinoma [113], glioblastoma [114], breast cancer [115], and other cancers [100].

### 3.3. The Antimalarial Drug Chloroquine

Chloroquine is a drug that was introduced to treat malaria in the mid-1940s, and it has been in continuous use for this since then. The mechanism of action in killing Plasmodia is by chlororoquine’s accumulation in the trophozoite’s acidic food vacuole, thereby preventing ingested hemoglobin degradation and/or by inhibiting the trophozoite’s heme polymerase [116,117,118]. Hydroxychloroquine and chloroquine are closely related and have similar uses in treating rheumatoid diseases such as rheumatoid arthritis, lupus, Sjögren’s, etc., as well as malaria. They both reduce mammalian lysosome function by increasing intralysosomal pH. They both result in clinically reduced inflammation-related cytokine production, inhibit cytokine production, and have a half-life of 40–60 days.

Their primary serious potential side effect of retinal injury is derived from their strong binding to melanin. For linguistic simplicity, the following discussion should be understood to refer to both when “chloroquine” is mentioned.

Much of the previous work on chloroquine as a cancer treatment adjunct generally [119,120,121,122,123], and specifically in OS [124], has been focused on chloroquine’s inhibition of autophagy. Indeed, chloroquine does this well and by known mechanisms [125]. This may be an important mode of OS growth inhibition, as these works outline. However, here, the focus is on chloroquine’s inhibition of inflammation-related cytokines and on its toll-like receptor-9 (TLR-9) inhibition.

TLR-9 is one of the many innate pathogen-recognizing systems cells have. It is activated by CpG DNA [126]. CpG DNA sites are cytosine-phosphate-guanine DNA sequence pairs that tend to occur grouped in localized DNA areas. Chloroquine inhibits TLR-9 signaling, as has been shown in diverse settings [127,128,129,130,131,132]. TLR-9 activation on normal osteoblasts by CpG DNA triggers, through intermediaries, nuclear NFkappaB activation and the expression of tumor necrosis factor-alpha (TNF) and macrophage-colony-stimulating factor (M-CSF) [133].

TLR-9 expression is increased in OS compared to normal osteoblasts [134]. The knockdown of TLR-9 inhibited OS growth and forced overexpression of TLR-9 increased OS growth in experimental models [135].

A similar pattern was seen in cholangiocarcinoma, where TLR-9 was overexpressed, yet it was absent in normal biliary tracts [136]. TLR-9 inhibition with chloroquine inhibited in vitro growth of cholangiocarcinoma and in xenografts [136]. The growth drive contributions of TLR-9 agonism have also been seen in the cells of breast [137] and esophagus [138] cancers.

### 3.4. The Antibiotic Dapsone

Dapsone is a sulfone antibiotic used to treat malaria, Hansen’s disease, tuberculosis, and other microbiological diseases. Similar to chloroquine, dapsone is effective for and used to treat rheumatological disease, particularly, rheumatoid arthritis [139,140,141].

IL-8 (also called CXCL8) is a neutrophil-attractant chemokine that signals via CXCR1 or CXCR2 receptors [142]. IL-8 is stored in neutrophils and the vascular endothelium.

Dapsone suppresses IL-8 synthesis [143,144,145]. Dapsone also suppresses neutrophil chemotaxis to IL-8 gradients [14,15,16,17,18,19,20,21,22,23,24,25,26,27,28,29,30,31,32,33,34,35,36,37,38,39,40,41,42,43,44,45,46,47,48,49,50,51,52,53,54,55,56,57,58,59,60,61,62,63,64,65,66,67,68,69,70,71,72,73,74,75,76,77,78,79,80,81,82,83,84,85,86,87,88,89,90,91,92,93,94,95,96,97,98,99,100,101,102,103,104,105,106,107,108,109,110,111,112,113,114,115,116,117,118,119,120,121,122,123,124,125,126,127,128,129,130,131,132,133,134,135,136,137,138,139,140,141,142,143,144,145,146,147,148]. Il-8 is an active participant in the joint-destructive inflammation of rheumatoid arthritis [149]. It is thought that dapsone reduces rheumatoid arthritis activity by reducing Il-8 and Il-8-mediated neutrophil accumulations. Dapsone also reduces mortality associated with COVID-19 and acute adult respiratory syndromes of any other origin by this same mechanism of action [150,151]. Neutrophil function is essential for life, but overly exuberant neutrophil function potentially creates tissue destruction and tumor trophic functions.

Exosomes from OS cells signal otherwise normal lung resident fibroblasts to secrete IL-8, facilitating the formation of the pulmonary metastases characteristic of OS [152,153]. IL-8 also forms one of the factors that trigger single OS cells to avoid anoikis [152,154]. OS patients have increased circulating IL-8 levels proportional to increased tumor burden [155,156,157,158,159]. Dogs with OS also have shown increased circulating IL-8 compared to normal dogs [160].

An interesting point about metastasis in cancer generally, and in OS specifically, is the extreme rarity of metastasis considering the millions of viable circulating tumor cells. There is experimental evidence in OS that the subset of circulating OS cells with greater IL-8 synthesis are the subset that can establish metastasis [152,155,159,161]. IL-8 signaling at CXCR1 is a cisplatin cytotoxicity resistance factor in OS [161].

**Figure 2:**
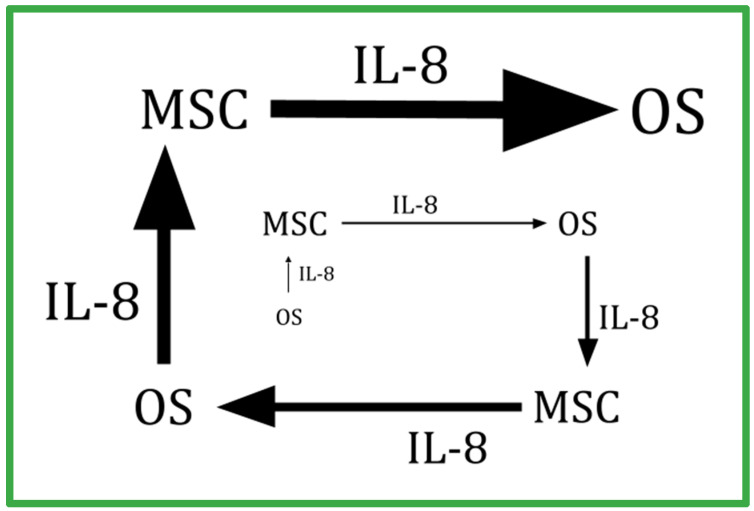
Schematic of the amplifying feedback loop between nonmalignant mesenchymal cells and malignant osteosarcoma (OS) cells showing the OS-secreted IL-8 that stimulates stromal mesenchymal stem cells (MSC) that also synthesize IL-8 and are stimulated to make more IL-8 that, in turn, stimulates OS cells to make more IL-8. A similar IL-8-centered feedback loop exists between circulating neutrophils and a tumor’s endothelium has been described in other cancers (refs. [162,163,164]). Dapsone, by decreasing IL-8 and blunting cells’ responses to IL-8, interrupts this amplifying feedback loop.

In 2018, Kawano et al. provided evidence that an amplifying IL-8 feedback loop exists between OS cells and stroma, where the OS release of IL-8 triggers stroma cells’ synthesis of IL-8, which then triggers the OS cell to make more IL-8 [162]. This positive feedback loop is diagrammed in Figure 2. This work has been supported by the work of others [163]. Of great interest is that this same IL-8-amplifying feedback loop has been described as occurring in the generation of the erlotinib skin rash and its inhibition by dapsone [164].

There are, however, dozens or hundreds of additional attributes beyond IL-8 that favor or do not favor an OS cell’s ability or inability to establish metastasis.

### 3.5. The Alcoholism Treatment Drug Disulfiram

Disulfiram is a drug that has been used since the 1950s to treat alcoholism by making ethanol ingestion highly unpleasant. It is a potent inhibitor of all the isoforms of aldehyde dehydrogenase (ALDH). Disulfiram thereby stops ethanol metabolism at the acetaldehyde stage. Flushing, hypotension, malaise, nausea, and vomiting ensue due to acetaldehyde accumulation if ethanol is consumed.

The basic rationale for adding disulfiram to OS treatment is based on the association between high ALDH expression in individual cells and those cells having stem cell attributes. This holds true for the stem cell subpopulations within common cancers [165,166], including throughout the sarcomas [167] and, specifically, in OS [168,169,170,171,172,173]. There are currently over 30 open studies on disulfiram as an adjuvant treatment in various cancers that were all based on that association (clinicaltrials.gov).

Recent works have continued to support an adjunctive cancer growth suppression role for disulfiram [174,175,176,177,178].

### 3.6. The Antifungal Drug Itraconazole

Itraconazole is a broad-spectrum antifungal agent that has been used clinically since the late 1980s. It is commonly used today for minor illnesses such as onychomycosis [179], and for serious fungal infections [180], or as an antifungal prophylaxis for the immunosuppressed [181,182,183].

Itraconazole inhibits the function of several cell physiological systems that are known to facilitate or drive growth across a variety of cancers [184]. Specifically, itraconazole has inhibited Hedgehog (Hh) signaling [185], O-glycosylation [186], the drug efflux pump P-gp [187], and 5-lipoxygenase 5-LO [188].

Specifically in OS, Hh signaling drives increased growth vigor. Hh inhibition retards growth, and higher Hh expression is associated with shorter OS survival, increased radioresistance, and chemotherapy cytotoxicity resistance [189,190,191,192,193,194,195,196,197,198,199,200,201].

Clinically, survival benefit from adjunctive use of itraconazole, has been shown in several human cancers, including colon cancer [202], pancreatic ductal adenocarcinoma [203], gastric cancer [204], non-small-cell lung cancer [205], and epithelial ovarian cancer [206]. A retrospective analysis of patients with acute lymphoblastic and acute myelogenous leukemia receiving daunorubicin where itraconazole was used as antifungal prophylaxis during the neutropenic nadir showed better remission rates than those not receiving itraconazole [207]. In vitro, intracellular levels of daunorubicin have increased proportionately as itraconazole levels rose from 0.5 to 5.0 microg/mL [208]. Experimental (non-marketed) Hh inhibitors have impaired OS growth in preclinical OS model systems [209].

Clinically achieved levels of itraconazole block VEGF binding to VEGFR2. This was traced to defective receptor trafficking, which, in turn, was secondary to the itraconazole-mediated defective glycosylation of VEGFR2. Itraconazole disrupts the orderly formation of mammalian outer cell surface lipid rafts and, therefore, tends to inhibit cytotoxic mechanisms that depend on target molecule expression on/in lipid raft concentrated domains [210,211]. Itraconazole has significantly inhibited breast cancer resistance protein (BCRP), P-gp, and other drug efflux pumps [212,213,214,215]. By this mechanism, itraconazole has lowered resistance to topotecan [216], doxorubicin, and etoposide [217].

Recent works have continued to support the potential for itraconazole to inhibit cancer growth [218,219,220,221].

### 3.7. The Anti-Diabetes Drug Linagliptin

The gliptins are a group of related drugs marketed to treat type 2 diabetes, and they include alogliptin, anagliptin, evogliptin, gemigliptin, linagliptin, omarigliptin, sitagliptin, teneligliptin, trelagliptin, and vildagliptin. They all inhibit dipeptidyl peptidase-4 (DPP4, also termed CD26) [222]. DPP4 is a dimeric serine exopeptidase that cleaves at the penultimate proline or alanine from the N-terminus of a polypeptide. The cleaved polypeptide usually, but not always, loses signaling activity. DPP-4 is found in both soluble and membrane-bound forms. The DPP-4 inhibitor linagliptin, clinically marketed to treat type 2 diabetes, has inhibited two OS cell lines in vitro [223]. However, a concentration of 50 microM was used to show that. This raises uncertainty about the clinical potential of linagliptin in OS. Increased DPP4 activity has indirectly promoted bone resorption and inhibits bone formation [224].

RUNX2 is a transcription factor crucial for multipotent mesenchymal cells’ differentiation into immature osteoblasts. OS cases with greater RUNX2 overexpression have worse prognosis [225,226,227]. Runx2 must form a heterodimer, thereby to acquire DNA binding and to mediate osteoblast maturation. DPP4 is overexpressed in OS tissues compared to corresponding normal bone tissues, and greater overexpression is associated with shorter survival [228,229].

**Figure 3:**
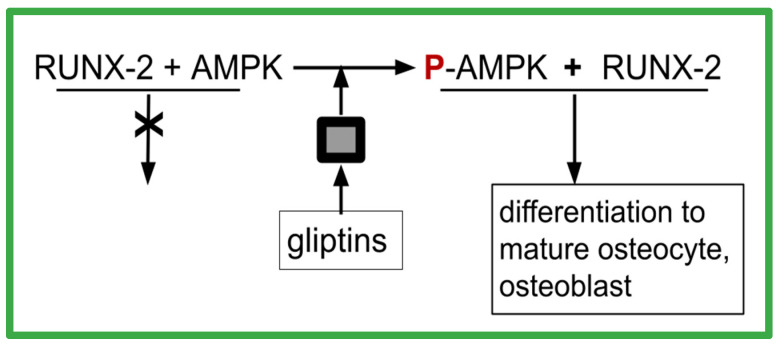
How gliptins allow RUNX-2 to function as differentiation factor. The black box indicates missing steps between the presence of a gliptin and the phosphorylation of AMPK. The intermediates are unknown.

As diagrammed in Figure 3, gliptin DPP-4 inhibitors result in increasing AMPK phosphorylation. This is one of the osteoblast maturation pathways of gliptins. Gemigliptin has increased the level of P-AMPK and Akt phosphorylation in human umbilical vein endothelial cells and in macrophages [230]. Consequent to that, gemigliptin has also reduced the levels of LPS-induced vascular cell adhesion molecule-1 (VCAM-1), E-selectin, TNF, monocyte chemoattractant protein-1 (MCP-1), interleukin-1 beta, and IL-6 [230]. In mice fed a high fat diet, linagliptin plus metformin reversed the elevated TNF and IL-1 beta levels that are otherwise generated by this diet [231]. Other DPP-4-inhibiting gliptins, such as omarigliptin [232], anagliptin [233], and trelagliptin [234], have promoted the in vitro maturation of osteoblasts. Omarigliptin has also enhanced the conversion of AMPK to phosphorylated AMPK (P-AMPK) in vitro in renal glomerular cells [235]. Other gliptins have also mediated AMPK phosphorylation [236,237,238,239,240].

P-AMPK is required for RUNX-2 to function as a differentiation transcription factor, as depicted in Figure 3. This might account for the seemingly paradoxical situation where OS has excess RUNX-2 yet is deficient in differentiation to mature well-behaved osteoblasts. The excess DPP-4 also characteristic of OS prevents the differentiation function of RUNX-2 by preventing the P-AMPK necessary for RUNX-2 to function properly. A second indicator supporting such a conjecture was the observation from large meta-analyses of clinical trials of DPP-4 inhibitors that showed 40% reductions in fractures in those on DPP-4 inhibitors, which was indicative of improved osteoblast function [241].

MC3T3-E1 cells are a commercial murine cell line that can differentiate into osteoblasts and osteocytes. The addition of saxagliptin in vitro increased osteopontin (OPN), RUNX-2, and type I collagen in MC3T3-E1 cells and enhanced their differentiation into mature osteoblasts [242]. This differentiation mediation was dependent on RUNX-2 and on saxagliptin’s mediation of the conversion of AMPK to phosphorylated AMPK (P-AMPK) [232,242]. Another gliptin DPP-4 inhibitor, anagliptin, has also increased the differentiation of MC3T3-E1 into mineralized osteoblasts via the activation of RUNX2 [233].

The above references can be summarized as follows:

- OS overexpress RUNX-2, yet the malignant clone fails to mature
- OS overexpress DPP-4
- P-AMPK is required for RUNX-2 to function in osteoblast maturation
- gliptins promote AMPK phosphorylation

From this, we can then hypothesize that a core feature of OS is differentiation (maturation) failure due to RUNX-2 function failure due to DPP-4-mediated local unavailability of the required co-factor P-AMPK. This is schematically depicted in Figure 3.

If linagliptin or any other DPP-4 inhibitor is used during the course of OS, an appropriate PET scan should be completed before starting treatment, and then again after 20–40 days on the DPP-4 inhibitor. This requirement is due to elements of uncertainty about the role of DPP-4, its inhibition, and malignant growth [243].

### 3.8. The Antihypertension Drug Propranolol

Propranolol is the first beta adrenergic receptor blocking drug to be marketed. Introduced to clinical practice in the 1960s, it continues to be in common use for treating hypertension, migraines, or pediatric hemangioma [244].

Although little has been published on this aspect of propranolol, a pre-performance beta-adrenergic blockade with propranolol is in wide use in musicians, actors, public speakers, etc. [245,246,247].

Propranolol reduces the physical impediments to music performances (e.g., sweating, dry mouth, and hand tremors) without reducing mental acuity, performance capability, or emotional components. This reduction in the physical signs of sympathetic overdrive is of relevance to propranolol’s intended use during cancer and, specifically, OS treatment.

Propranolol’s sympathetic drive reduction by blocking the beta-adrenergic receptor as a cancer treatment adjunct, including in sarcomas and OS specifically, occurs through several putative mechanisms [247,248,249,250,251,252,253,254]. The primacy of any one of these paths has not been established.

Beta-adrenergic signaling is one of the cancer-related drivers of the elevated numbers of myeloid derived suppressor cells characteristically seen in most of the common cancers [255,256,257,258,259]. Beta-adrenergic signaling particularly contributes to conditioning lung tissues to facilitate metastasis establishment [260,261].

Normal bone is innervated by the sympathetic nervous system acting on beta-adrenergic receptors [262,263]. OS tissues overexpress the beta-adrenergic receptor [264]. Trabecular and cortical bone density is increased by a beta-adrenergic blockade and conversely decreased by an unchecked beta-adrenergic drive [265,266,267,268]. Specifically in OS, propranolol has retarded growth and increased cisplatin’s growth retardation in murine and canine OS models [269,270,271].

### 3.9. The Psychiatric Drug Quetiapine

Quetiapine is a drug used in psychiatry to reduce the signs and symptoms of psychosis, to augment an inadequate response to antidepressant drugs, and to stop disrupted sleep–wake cycles. It is also eminently safe to use in non-psychiatric populations.

Normal mammalian bones are constantly being absorbed by osteoclasts and resynthesized by osteoblasts throughout life, a process called remodeling [272]. Osteoclasts differentiate from hematopoietic progenitors, and osteoblasts differentiate from mesenchymal stem cells.

Receptor activator for nuclear factor-kappa B ligand (RANKL) and its decoy receptor osteoprotegerin (OPG), a soluble glycoprotein 60-kDa monomer, are expressed on osteoblasts while RANK is expressed on osteoclasts. OPG, the soluble RANKL decoy receptor, reduces RANKL-driven osteoclastogenesis by binding RANKL, limiting RANKL signaling to RANK.

The RANK/RANKL/OPG system lies at the core of osteosarcoma. In OS, this RANK–RANKL process participates in creating the characteristic patchy areas of bone resorption. Bone metastasis is common in other cancers where the RANK–RANKL system also is a core mediating system in perimetastasis bone destruction [273,274,275].

The immunohistochemical localization of RANK-positive cells in human OS biopsy tissues tend to be seen in osteoclasts at the tumor–bone interface and in intratumoral osteoclasts and in the myeloid osteoclast precursors more than in the OS cells themselves. OS cells themselves express RANKL [276]. RANK–RANKL signaling mediates the trophic function of OS stromal cells, facilitating or permitting the malignant cells themselves to thrive [276,277,278,279,280,281]. Larger total OS tumor tissue RANK expression has been associated with shorter OS survival.

RANKL binds to osteoclast the outer membrane receptor RANK on immature osteoclast precursors. This triggers widespread changes, including NFkB nuclear translocation, mitogen-activated protein kinase activations, activating protein 1 (AP-1), activating the nuclear factor of activated T cells 1 (NFAT-1), and activating phosphatidylinositol 3-kinase (PI3K)/Akt, ending in multinucleated, active osteoclasts. This is a key factor in immature preosteoclast cell differentiation and activation.

High-dose quetiapine (100 microM) is nontoxic to osteoclasts in vitro, yet even lower doses (25 microM) can suppress RANK–RANKL driven in vitro osteoclast maturation and function [282]. In a breast cancer metastasis murine model, quetiapine inhibited perimetastasis bone destruction [282]. Quietapine also inhibited RANKL-induced osteoclast differentiation in a mouse macrophage cell line with no sign of general cytotoxicity [282].

Mature oligodendrocytes make myelin and oligodendrocyte progenitor cells do not. A series of preclinical studies have shown that quetiapine stimulates the maturation of oligodendrocyte progenitor cells and their myelin synthesis [283,284,285,286,287,288]. Reflecting this improved myelination and oligodendrocyte maturation, a human study on mania showed that a year of quetiapine treatment repaired the impaired functional connectivity of brain regions characteristic of untreated mania [289].

Heterotopic hepatocellular carcinoma tumors grew more slowly in quetiapine treated mice, but the proximate mediating event of the growth inhibition was unknown [290].

We have no direct evidence that quetiapine will enhance malignant OS cell maturation or slow OS growth, but the above evidence allows a plausible pathway by which quetiapine might do this by depriving OS of the RANK–RANKL signaling that is active in the trophic nonmalignant cells within a tumor mass.

## 4. TICO

The neutrophil-to-lymphocyte ratio (NLR) is the ratio of the number of neutrophils compared to the number of lymphocytes in peripheral blood. A higher than normal NLR is a common finding across the common cancers [291,292,293,294]. The consensus finding across these studies is that as this ratio progressively exceeds 3:1, the prognosis decreases.

Fourteen independent clinical studies conducted specifically on OS have shown that the higher an NLR becomes, the shorter the survival becomes [294,295,296,297,298,299,300,301,302,303,304,305,306]. The extraordinary nature of this association of a higher NLR with a shorter survival in adult and pediatric OS should be fully appreciated. There is no other finding, in the entire body of oncology knowledge, that is so uniformly found across all the common cancers, generally, and as above, in OS, specifically.

TICO is a repurposed drug regimen designed to lower the NLR [44]. Given the strong association of a higher NLR with shorter survival in OS, the TICO regimen should be considered as a treatment adjunct in OS.

The TICO regimen has marshaled past research showing NLR reductions after individual exposure to tadalafil, isotretinoin, colchicine, and omega-3. Therefore, TICO proposes using all four during cancer treatment to improve prognosis by pharmacologically lowering NLRs. However, TICO has not yet been proven to be safe or effective.

## 5. Discussion and Conclusions

This paper discusses several pathogenic mediators in OS and potential already marketed repurposed drugs to inhibit these systems, but it is important to remember that many more pathogenic physiological derangements are present in OS beyond those discussed here [196,307,308,309,310]. As with other deadly cancers, the malignant behavior of OS cells involves deranged activation and/or pathological drop-out and/or the pathologically active participation of dozens (or hundreds) of cell-signaling systems. The drugs and the pathology-driving systems they inhibit, as mentioned in this paper on OSR9, are the systems we might currently be able to easily inhibit. How many pathology-driving systems must we inhibit to stop OS growth? We do not know, but treatment with OSR9 is a start.

There have been no controlled trials of the OSR9 drugs. For the reasons listed in Section 2.1 and Section 2.2 above, we should not expect meaningful effects from any one or two drug additions to the current treatments. Hence, CUSP9v3 and the current OSR9 have nine or more drugs.

A current trial of adjuvant hydroxychloroquine with docetaxel and gemcitabine (NCT03598595) was started in June of 2023. We may not achieve positive results in this trial. The need for a many-drug regimen and the reasons why these many drugs must be taken from general medicine, as outlined in Section 2 of this paper, represent the thinking of a new school of oncology. Although currently still a minority among oncologists and researchers, this movement is gaining adherents. OSR9 is a further contribution to this trend.

As things currently stand in 2023, and given the likely outcome of metastatic OS and the likely benign side effects expected from the OSR9 medicines, this regimen might be worth a small pilot study.

## Figures and Tables

**Table 1:** Overview of the nine OSR9 drugs (references are in the text). #, used in studies on the clinical treatment of glioblastoma; ALDH, aldehyde dehydrogenase; CA, carbonic anhydrase; COX, cyclooxygenase; GLP-1, glucagon-like peptide-1; DPP-4, dipeptidyl peptidase-4; Hh, Hedgehog; 5-LO, 5-lipoxygenase; RANK, receptor activator for nuclear factor-kappa B; RANKL, receptor activator for nuclear factor-kappa B ligand; TLR, toll-like receptor.

Drug	Common Use	Targets in OSR9
aprepitant #	nausea	NK-1 receptor
celecoxib #	analgesia	CA-2-9 and COX-2
chloroquine	malaria and rheumatoid arthritis	TLR-9
dapsone	malaria and Hansen’s disease	IL-8 and neutrophils
disulfiram #	alcoholism	ALDH
itraconazole #	fungal infections	Hh and 5-LO
linagliptin	diabetes	GLP-1 and DPP-4
propranolol	hypertension	beta adrenergic receptor
quetiapine	psychosis	RANK-RANKL

## Data Availability

All data has been presented in the paper.
